# Prognosis of post-neoadjuvant therapy patients who underwent immediate breast reconstruction: a SEER-based, propensity-matched study

**DOI:** 10.1007/s12282-023-01489-8

**Published:** 2023-07-28

**Authors:** Jiahao Pan, Liying Peng, Xiuwen Tong, Xipei Chen, Xinyun Xu, Jian Zhang

**Affiliations:** 1 Department of General Surgery, Changzheng Hospital of the Second Military Medical University, 415 Fengyang Road, Shanghai, 200000 China; 2 Department of Hematology, Changzheng Hospital of the Second Military Medical University, 415 Fengyang Road, Shanghai, 200000, China

**Keywords:** Breast cancer, Neoadjuvant therapy, Immediate breast reconstruction, SEER Program

## Abstract

**Objective:**

The application of immediate breast reconstruction (IBR) for post-neoadjuvant therapy (NAT) patients was controversial. The aim of this study was to investigate the long-term survival outcomes of IBR for these patients.

**Methods:**

Data between January 2010 and November 2017 were extracted from the Surveillance, Epidemiology, and End Results (SEER) database. Propensity score matching (PSM) was performed to reduce the influence of confounding factors between the mastectomy alone group (MA) and the mastectomy with IBR group (IBR). The rates of 5 year breast cancer-specific survival (BCSS) were compared by Kaplan–Meier curves with log-rank test.

**Results:**

The IBR was associated with improved 5-year BCSS in the IBR group before PSM (88.5 vs. 79.1%, *P* < 0.001). The proportion of IBR increased from 21.5% in 2010 to 28.2% in 2017. After PSM, a total of 9,610 patients were enrolled for survival analysis (4,805 in each group). In the complete response (CR) group, the 5-year BCSS rates did not differ (93.4 vs. 95.6%, *P* = 0.16). In the non-CR group, the 5-year BCSS rate was higher in patients who received IBR (82.5% 79.4%, *P* = 0.034).

**Conclusion:**

In general, the application of IBR among post-NAT patients has steadily increased from 2010 to 2017. In the CR group, survival outcomes of post-NAT patients who received IBR were similar to those who received mastectomy alone. In the non-CR group, IBR was associated with potential survival benefits. More studies are expected to validate our findings.

## Introduction

Neoadjuvant therapy (NAT) plays a vital role in the treatment of breast cancer, which includes neoadjuvant chemotherapy (NACT), neoadjuvant radiotherapy (NART), endocrine therapy and targeted therapy. NACT was originally recommended to treat locally advanced breast cancer, because of the effectiveness of downstaging tumors to facilitate surgery, as well as facilitating breast conservation in such cases [1, 2]. To date, the indications of NACT have gradually expanded to triple-negative, human epidermal growth factor receptor 2 positive (HER2 +), or early-stage breast cancer [3, 4]. Meanwhile, during the treatment, NART was typically used in conjunction with NACT to treat initial inoperable breast cancer [5]. Furthermore, drug sensitivity of breast cancer patients could be studied during the treatment to guide subsequent therapy to improve outcomes.

Mastectomy followed by immediate breast reconstruction (IBR), involving autologous flap reconstruction and implant-based reconstruction, has gained popularity among patients. In comparison to delayed breast reconstruction, IBR offers better cosmetic outcomes, more positive psychological impact, and only one operation with less financial burden [6–8]. However, the issue of IBR for post-NAT patients was controversial, with different conclusions being reached by different studies. Gouy et al. confirmed that IBR after NACT did not significantly affect the local relapse-free or distant disease-free survival [9]. Wu et al. also demonstrated the comparable long-term oncologic outcomes of IBR with nipple-sparing or skin-sparing mastectomy to conventional mastectomy alone [10]. However, according to a previous study which was based on data from the Korean Breast Cancer Society, IBR following mastectomy was associated with worse prognoses than mastectomy alone in non-pCR patients with advanced clinical stages of cancer [11]. Meanwhile, a number of studies were concerned that NACT may increase local recurrence risk after breast-conserving surgery (BCS) [12, 13]. In addition, discrepancies in complication rates across studies hindered decision-making about whether to receive IBR in the setting of NAT [14–16].

In view of the personal preference of patients to breast reconstruction, it is difficult to conduct a randomized controlled trial to investigate IBR’s outcomes. Meanwhile, prior studies, mainly retrospective single-center studies, provided insufficient evidence. Consequently, we used the SEER database to perform this large population-based research that investigate the long-term prognosis of post-NAT patients who underwent IBR.

## Materials and methods

### Population

Approximately 28% of the national population was included in SEER, which collected data from 18 cancer registries across the United States. Using the SEER*Stat 8.4.0.1, 20–80 years old women who were diagnosed with primary M0 stage breast cancer and received neoadjuvant therapy between January 2010 and November 2017 were initially identified. Exclusion criteria included: (1) more than one malignant tumor; (2) T0 or Tis stage; (3) bilateral breast cancer; (4) not receive surgery or with unknown surgical information. Finally, all the eligible patients were divided into breast-conserving group (BCS, surgery codes 19–24), mastectomy alone group (MA, surgery codes 40–42, 50–52, 60–62, 70–72) and mastectomy with IBR group (IBR, surgery codes 30, 43–49, 53–59, 63–69, 73–75).

### Variables and definitions

An unmarried status was defined as divorced, separated, single, domestic partner, or widowed at the time of diagnosis. Grade 1–4 were defined as well, moderately, poorly and undifferentiated in ICD-O-2, respectively. Histologic type included infiltrating duct carcinoma (IDC, ICD-O-3 code 8500), infiltrating lobular carcinoma (ILC, ICD-O-3 code 8520), infiltrating duct and lobular carcinoma (ICD-O-3 code 8500) and others. Contralateral breast mastectomy (CPM) and reconstruction type were based on surgery codes. The order of radiotherapy and systemic therapy was preoperative, intraoperative, postoperative or perioperative. Both therapies were reduced to given and not given for analysis. According to the SEER Program Coding and Staging Manual 2022 [17], response to neoadjuvant therapy (RNT) was recorded based on the surgical pathology report. Complete pathological response (CR) denoted the absence of the primary tumor. A partial response (PR) was defined as a decrease in tumor size/extent and evidence of tumor regression. No definite response (NR) referred to a tumor's size or extent showing no change at all or showing considerable remaining invasive malignancy. Unknown response indicated that NAT was completed but there was no documented treatment response in the surgical pathology report. Non-CR in this work included PR and NR. The cutoff date for this research was November 2017. The time from diagnosis to death related to breast cancer was the definition of BCSS.

### Propensity score matching

Propensity score matching (PSM) was carried out in a 1:1 ratio in the comparison between the MA group and the IBR group using the R package "MatchIt", with the parameters of the "nearest" method and a caliper of 0.02. This was done to lessen the impact of confounding factors on prognosis. Age, year of diagnosis, race, marital status at diagnosis, grade, histology, T stage, N stage, CPM, RNT, radiation, systemic therapy, estrogen receptor (ER) status, progesterone receptor (PR) status, HER2 status, and median household income were the baseline characteristics used for matching.

### Statistical analysis

All analyses were performed using R software (version 4.2.1; http://www.r-project.org/). To compare normally distributed data that were expressed as mean ± standard deviation (x ± s), Student's t-test was utilized. Non-normally distributed data that were reported as median (interquartile range, IQR) were analyzed using the Mann–Whitney *U* test. Categorical data was presented as n (%) and was then examined using the Chi-square test or the Fisher's exact test. Kaplan–Meier method with log-rank test was used to assess 5 year BCSS. The statistical significance levels were both two-sided and set at *P* < 0.05.

## Results

**Fig. 1:**
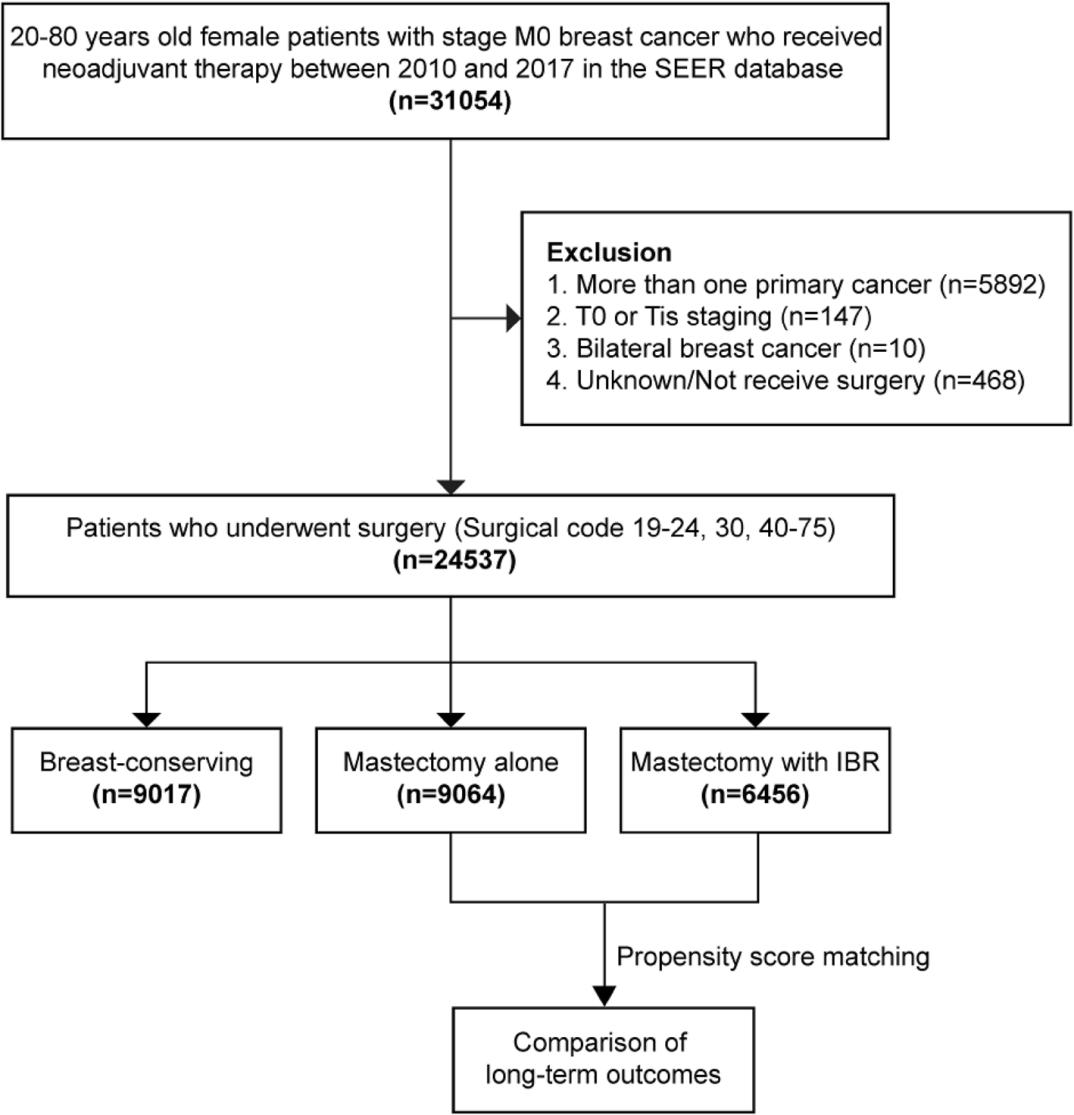
Work Flow Diagram. SEER database, Surveillance, Epidemiology, and End Results (SEER) database; IBR, immediate breast reconstruction

We obtained information on 31,054 female patients aged 20 to 80 who had M0 stage breast cancer and had undergone NAT between 2010 and 2017. In Fig. 1, the screening flow is displayed. A total of 24,537 eligible patients were eventually enrolled, of whom 9,017 underwent breast-conserving surgery, 9,064 had a mastectomy alone and 6,456 underwent mastectomy with IBR. The MA group and the IBR group were further compared.

### Demographic features

**Fig. 2:**
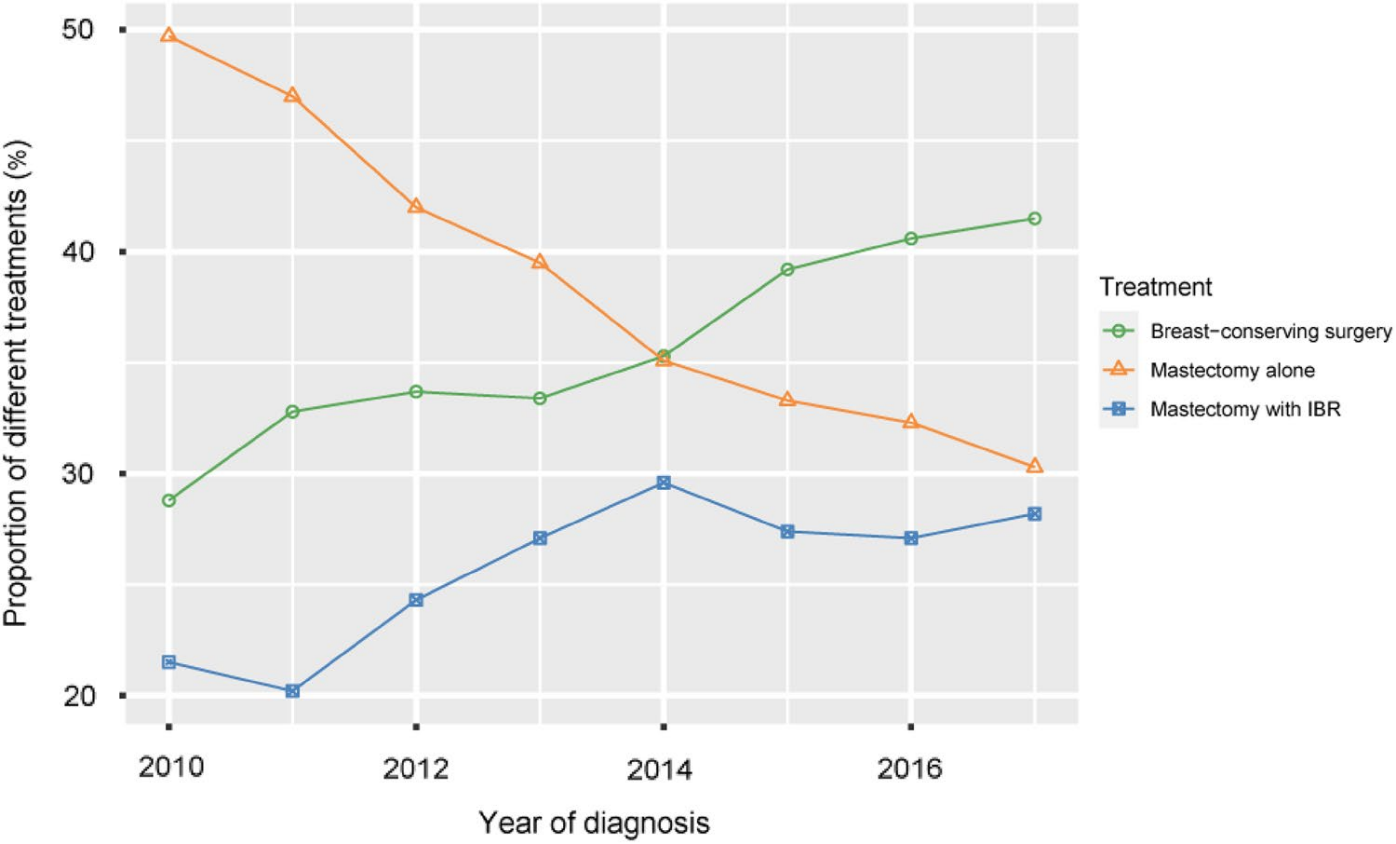
Trends in applications of breast-conserving, mastectomy alone and mastectomy with immediate breast reconstruction from 2010 to 2017. IBR, immediate breast reconstruction

Trends in the application of the three treatments are shown in Fig. 2. Generally, the proportion of post-NAT patients who underwent either breast-conserving surgery (28.8 to 41.5%) or mastectomy with IBR (21.5 to 28.2%) was increasing from 2010 to 2017, and the proportion of mastectomy alone was declining (49.7 to 30.3%). Table 1 reports the comparison of the MA group and the IBR group. Mastectomy with IBR was associated with younger, white or married patients (*P* < 0.001, respectively). In addition, patients in both groups mostly earned between $50,000 and $70,000, while the patients with income higher than $70,000 were more in the IBR group (*P* < 0.001).

**Table 1:** Demographic and baseline characteristics of the mastectomy alone group and the immediate breast reconstruction group

Characteristics	Before PSM			*P* value^*^	After PSM			*P* value^*^
	Total (N = 15,520) (%)	MA group (N = 9064) (%)	IBR group (N = 6456) (%)		Total (N = 9610) (%)	MA group (N = 4805) (%)	IBR group (N = 4805) (%)	
Age, n (%)				< 0.001				0.937
< 41	3495 (22.5)	1477 (16.3)	2018 (31.3)		2393 (24.9)	1203 (25.0)	1190 (24.8)	
41–60	8645 (55.7)	4947 (54.6)	3698 (57.3)		5776 (60.1)	2886 (60.1)	2890 (60.1)	
> 60	3380 (21.8)	2640 (29.1)	740 (11.5)		1441 (15.0)	716 (14.9)	725 (15.1)	
Year of diagnosis, n (%)				< 0.001				0.856
2010–2011	2894 (18.6)	2023 (22.3)	871 (13.5)		1555 (16.2)	780 (16.2)	775 (16.1)	
2012–2013	3319 (21.4)	2033 (22.4)	1286 (19.9)		2056 (21.4)	1041 (21.7)	1015 (21.1)	
2014–2015	4454 (28.7)	2430 (26.8)	2024 (31.4)		2901 (30.2)	1433 (29.8)	1468 (30.6)	
2016–2017	4853 (31.3)	2578 (28.4)	2275 (35.2)		3098 (32.2)	1551 (32.3)	1547 (32.2)	
Race, n (%)				< 0.001				0.935
White	11,385 (73.4)	6383 (70.4)	5002 (77.5)		7131 (74.2)	3579 (74.5)	3552 (73.9)	
Black	2288 (14.7)	1446 (16.0)	842 (13.0)		1394 (14.5)	689 (14.3)	705 (14.7)	
Others^a^	1741 (11.2)	1172 (12.9)	569 (8.8)		1019 (10.6)	505 (10.5)	514 (10.7)	
Unknown	106 (0.7)	63 (0.7)	43 (0.7)		66 (0.7)	32 (0.7)	34 (0.7)	
Marital status, n (%)				< 0.001				0.849
Married	9094 (58.6)	4900 (54.1)	4194 (65.0)		5879 (61.2)	2948 (61.4)	2931 (61.0)	
Unmarried	5884 (37.9)	3808 (42.0)	2076 (32.2)		3420 (35.6)	1706 (35.5)	1714 (35.7)	
Unknown	542 (3.5)	356 (3.9)	186 (2.9)		311 (3.2)	151 (3.1)	160 (3.3)	
Income, n (%)				< 0.001				0.726
< $50,000	2215 (14.3)	1567 (17.3)	648 (10.0)		1158 (12.1)	573 (11.9)	585 (12.2)	
$50,000–$70,000	7867 (50.7)	4681 (51.6)	3186 (49.3)		5015 (52.2)	2527 (52.6)	2488 (51.8)	
> $70,000	5438 (35.0)	2816 (31.1)	2622 (40.6)		3437 (35.8)	1705 (35.5)	1732 (36.0)	
Grade, n (%)				< 0.001				0.906
I	818 (5.3)	493 (5.4)	325 (5.0)		517 (5.4)	252 (5.2)	265 (5.5)	
II	5065 (32.6)	2938 (32.4)	2127 (32.9)		3198 (33.3)	1593 (33.2)	1605 (33.4)	
III/IV	8696 (56.0)	5026 (55.5)	3670 (56.8)		5333 (55.5)	2681 (55.8)	2652 (55.2)	
Unknown	941 (6.1)	607 (6.7)	334 (5.2)		562 (5.8)	279 (5.8)	283 (5.9)	
Tumor location, n (%)				< 0.001				0.205
Nipple	42 (0.3)	30 (0.3)	12 (0.2)		23 (0.2)	15 (0.3)	8 (0.2)	
Central portion	800 (5.2)	530 (5.8)	270 (4.2)		438 (4.6)	225 (4.7)	213 (4.4)	
Upper-inner quadrant	1420 (9.1)	765 (8.4)	655 (10.1)		932 (9.7)	461 (9.6)	471 (9.8)	
Lower-inner quadrant	626 (4.0)	364 (4.0)	262 (4.1)		388 (4.0)	203 (4.2)	185 (3.9)	
Upper-outer quadrant	5133 (33.1)	2987 (33.0)	2146 (33.2)		3210 (33.4)	1648 (34.3)	1562 (32.5)	
Lower-outer quadrant	1026 (6.6)	549 (6.1)	477 (7.4)		660 (6.9)	318 (6.6)	342 (7.1)	
Axillary tail	74 (0.5)	47 (0.5)	27 (0.4)		44 (0.5)	26 (0.5)	18 (0.4)	
Overlapping lesion	3548 (22.9)	2042 (22.5)	1506 (23.3)		2215 (23.0)	1069 (22.2)	1146 (23.9)	
Non-specific	2851 (18.4)	1750 (19.3)	1101 (17.1)		1700 (17.7)	840 (17.5)	860 (17.9)	
Laterality, n (%)				0.146				0.403
Left	7859 (50.6)	4635 (51.1)	3224 (49.9)		4878 (50.8)	2460 (51.2)	2418 (50.3)	
Right	7661 (49.4)	4429 (48.9)	3232 (50.1)		4732 (49.2)	2345 (48.8)	2387 (49.7)	
Histology, n (%)				< 0.001				0.877
IDC	12,568 (81.0)	7187 (79.3)	5381 (83.3)		7877 (82.0)	3945 (82.1)	3932 (81.8)	
ILC	922 (5.9)	567 (6.3)	355 (5.5)		581 (6.0)	283 (5.9)	298 (6.2)	
IDC mixed with ILC	631 (4.1)	378 (4.2)	253 (3.9)		381 (4.0)	195 (4.1)	186 (3.9)	
Other	1399 (9.0)	932 (10.3)	467 (7.2)		771 (8.0)	382 (8.0)	389 (8.1)	
Stage^b^, n (%)				< 0.001				0.622
I	1222 (7.9)	497 (5.5)	725 (11.2)		823 (8.6)	395 (8.2)	428 (8.9)	
II	7062 (45.5)	3562 (39.3)	3500 (54.2)		4870 (50.7)	2443 (50.8)	2427 (50.5)	
III	6862 (44.2)	4742 (52.3)	2120 (32.8)		3710 (38.6)	1867 (38.9)	1843 (38.4)	
Unknown	374 (2.4)	263 (2.9)	111 (1.7)		207 (2.2)	100 (2.1)	107 (2.2)	
T staging^b^, n (%)				< 0.001				0.89
1	2474 (15.9)	1171 (12.9)	1303 (20.2)		1675 (17.4)	821 (17.1)	854 (17.8)	
2	6563 (42.3)	3409 (37.6)	3154 (48.9)		4445 (46.3)	2226 (46.3)	2219 (46.2)	
3	3608 (23.2)	2172 (24.0)	1436 (22.2)		2377 (24.7)	1197 (24.9)	1180 (24.6)	
4	2520 (16.2)	2060 (22.7)	460 (7.1)		920 (9.6)	467 (9.7)	453 (9.4)	
Unknown	355 (2.3)	252 (2.8)	103 (1.6)		193 (2.0)	94 (2.0)	99 (2.1)	
N staging^b^, n (%)				< 0.001				0.721
0	4764 (30.7)	2275 (25.1)	2489 (38.6)		3147 (32.7)	1551 (32.3)	1596 (33.2)	
1	6978 (45.0)	4158 (45.9)	2820 (43.7)		4455 (46.4)	2247 (46.8)	2208 (46.0)	
2	2242 (14.4)	1530 (16.9)	712 (11.0)		1256 (13.1)	641 (13.3)	615 (12.8)	
3	1454 (9.4)	1040 (11.5)	414 (6.4)		712 (7.4)	347 (7.2)	365 (7.6)	
Unknown	82 (0.5)	61 (0.7)	21 (0.3)		40 (0.4)	19 (0.4)	21 (0.4)	
CPM, n (%)				< 0.001				0.698
No	9093 (58.6)	6202 (68.4)	2891 (44.8)		5118 (53.3)	2569 (53.5)	2549 (53.0)	
Yes	6427 (41.4)	2862 (31.6)	3565 (55.2)		4492 (46.7)	2236 (46.5)	2256 (47.0)	
Reconstruction type, n (%)				< 0.001				< 0.001
Not given	9064 (58.4)	9064 (100)	0 (0)		4805 (50.0)	4805 (100)	0 (0)	
Implant	2206 (14.2)	0 (0)	2206 (34.2)		1542 (16.0)	0 (0)	1542 (32.1)	
Tissue	1539 (9.9)	0 (0)	1539 (23.8)		1156 (12.0)	0 (0)	1156 (24.1)	
Implant with tissue	623 (4.0)	0 (0)	623 (9.7)		478 (5.0)	0 (0)	478 (9.9)	
Unknown	2088 (13.5)	0 (0)	2088 (32.3)		1629 (17.0)	0 (0)	1629 (33.9)	
RNT, n (%)				< 0.001				0.934
CR	4347 (28.0)	2284 (25.2)	2063 (32.0)		2798 (29.1)	1397 (29.1)	1401 (29.2)	
Non-CR^c^	7013 (45.2)	4437 (49.0)	2576 (39.9)		4156 (43.2)	2072 (43.1)	2084 (43.4)	
Unknown^d^	4160 (26.8)	2343 (25.8)	1817 (28.1)		2656 (27.6)	1336 (27.8)	1320 (27.5)	
Radiation, n (%)				< 0.001				0.623
Given	8711 (56.1)	5505 (60.7)	3206 (49.7)		5261 (54.7)	2643 (55.0)	2618 (54.5)	
After surgery	8510 (54.8)	5374 (59.3)	3136 (48.6)		5138 (53.5)	2579 (53.7)	2559 (53.3)	
Before surgery	92 (0.6)	66 (0.7)	26 (0.4)		53 (0.6)	30 (0.6)	23 (0.5)	
Both before and after	100 (0.6)	60 (0.7)	40 (0.6)		64 (0.7)	32 (0.7)	32 (0.7)	
Unknown sequence	9 (0.1)	5 (0.1)	4 (0.1)		6 (0.1)	2 (0.0)	4 (0.1)	
Not given	6809 (43.9)	3559 (39.3)	3250 (50.3)		4349 (45.3)	2162 (45.0)	2187 (45.5)	
Systemic therapy, n (%)				0.119				0.556
Given	15,478 (99.7)	9034 (99.7)	6444 (99.8)		9584 (99.7)	4790 (99.7)	4794 (99.8)	
After surgery	874 (5.6)	488 (5.4)	386 (6.0)		579 (6.0)	291 (6.1)	288 (6.0)	
Before surgery	6910 (44.5)	4154 (45.8)	2756 (42.7)		4218 (43.9)	2148 (44.7)	2070 (43.1)	
Both before and after	7692 (49.6)	4390 (48.4)	3302 (51.1)		4786 (49.8)	2350 (48.9)	2436 (50.7)	
Unknown sequence	2 (0.0)	2 (0.0)	0 (0)		1 (0.0)	1 (0.0)	0 (0)	
Not given	42 (0.3)	30 (0.3)	12 (0.2)		26 (0.3)	15 (0.3)	11 (0.2)	
Molecular subtype, n (%)				< 0.001				0.201
HR−/HER2−	3525 (22.7)	2022 (22.3)	1503 (23.3)		2161 (22.5)	1063 (22.1)	1098 (22.9)	
HR−/HER2 +	1852 (11.9)	1134 (12.5)	718 (11.1)		1129 (11.7)	586 (12.2)	543 (11.3)	
HR + /HER2−	6144 (39.6)	3702 (40.8)	2442 (37.8)		3790 (39.4)	1930 (40.2)	1860 (38.7)	
HR + /HER2 +	3475 (22.4)	1835 (20.2)	1640 (25.4)		2262 (23.5)	1094 (22.8)	1168 (24.3)	
Unknown	524 (3.4)	371 (4.1)	153 (2.4)		268 (2.8)	132 (2.7)	136 (2.8)	
ER status, n (%)				< 0.001				0.617
Negative	5829 (37.6)	3420 (37.7)	2409 (37.3)		3552 (37.0)	1790 (37.3)	1762 (36.7)	
Positive	9531 (61.4)	5527 (61.0)	4004 (62.0)		5985 (62.3)	2982 (62.1)	3003 (62.5)	
Borderline/Unknown	160 (1.0)	117 (1.3)	43 (0.7)		73 (0.8)	33 (0.7)	40 (0.8)	
PR status, n (%)				< 0.001				0.707
Negative	7773 (50.1)	4619 (51.0)	3154 (48.9)		4743 (49.4)	2384 (49.6)	2359 (49.1)	
Positive	7562 (48.7)	4302 (47.5)	3260 (50.5)		4793 (49.9)	2387 (49.7)	2406 (50.1)	
Borderline/unknown	185 (1.2)	143 (1.6)	42 (0.7)		74 (0.8)	34 (0.7)	40 (0.8)	
HER2 status, n (%)				< 0.001				0.715
Negative	9682 (62.4)	5735 (63.3)	3947 (61.1)		5957 (62.0)	2998 (62.4)	2959 (61.6)	
Positive	5338 (34.4)	2975 (32.8)	2363 (36.6)		3396 (35.3)	1680 (35.0)	1716 (35.7)	
Borderline/unknown	500 (3.2)	354 (3.9)	146 (2.3)		257 (2.7)	127 (2.6)	130 (2.7)	

*PSM* propensity score matching, *IDC* invasive ductal carcinoma, *ILC* invasive lobular carcinoma, *CPM* contralateral prophylactic mastectomy, *RNT* response to neoadjuvant therapy, *CR* complete response, *ER* estrogen receptor, *PR* progesterone receptor, *HER2* human epidermal growth factor receptor type 2

*The comparison between the MA group and the IBR group

^a^Others include American Indian/AK Native, Asian/Pacific Islander

^b^Stage, T staging and N staging are according to the American Joint Committee on Cancer Staging Manual 7th edition

^c^Non-CR includes partial response and no definite response

^d^Unknown-response is defined as reponse to treatment, but not noted if complete or partial

### Clinical and pathological characteristics

With the exception of laterality, every clinical and pathological characteristic between groups was statistically significant (Table 1). Grade III/IV (55.5% in the MA group and 56.8% in the IBR group) and IDC (79.3% in the MA group and 83.3% in the IBR group) made up the majority in each group. The IBR group related to more patients with T1-2 and N0 stage breast cancer, but fewer patients with T3-4 and N1-3 stage breast cancer. In the IBR group, CR was more prevalent (32.0 vs. 25.2%), whereas in the MA group, NR was more prevalent (10.4 vs. 7.2%, *P* < 0.001). The most prevalent breast subtype was HR + /HER2- (40.8% in the MA group and 37.8% in the IBR group), whereas HR-/HER2 + was the least common (12.5% in the MA group and 11.1% in the IBR group).

### Therapeutic characteristics

In the IBR group compared to the MA group, CPM was more prevalent (55.2% vs. 31.6%, *P* < 0.001). In the IBR group, implant-based reconstruction (34.2%) was the most common. In the both groups, systemic therapy was given to the vast majority of the patients. However, radiation was given to more patients in the MA group (60.7 vs. 49.7%, *P* < 0.001).

### Comparison of survival outcomes

**Fig. 3:**
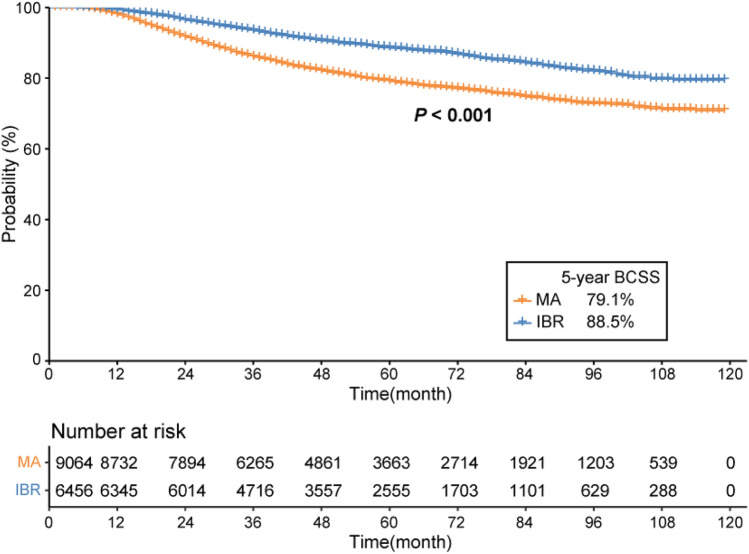
Breast cancer-specific survival rates of the MA versus IBR groups before propensity score matching. *BCSS* breast cancer-specific survival, *MA* mastectomy alone, *IBR* immediate breast reconstruction

**Fig. 4:**
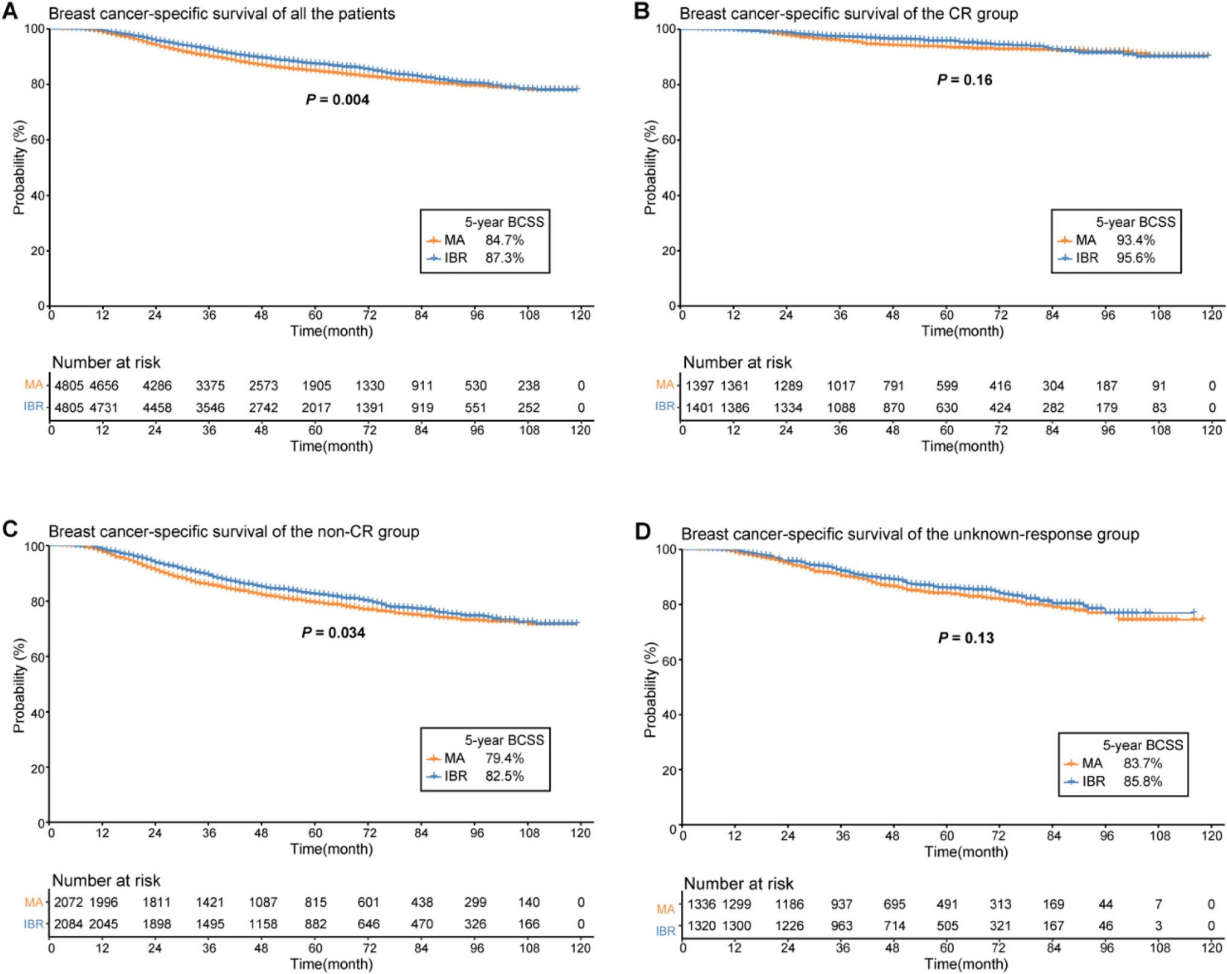
Breast cancer-specific survival rates of the MA versus IBR groups after propensity score matching. **A** All the patients; **B** The CR group; **C** The non-CR group; **D** The unknown-response group. *BCSS* breast cancer-specific survival, *CR* complete response, *MA* mastectomy alone, *IBR* immediate breast reconstruction

**Fig. 5:**
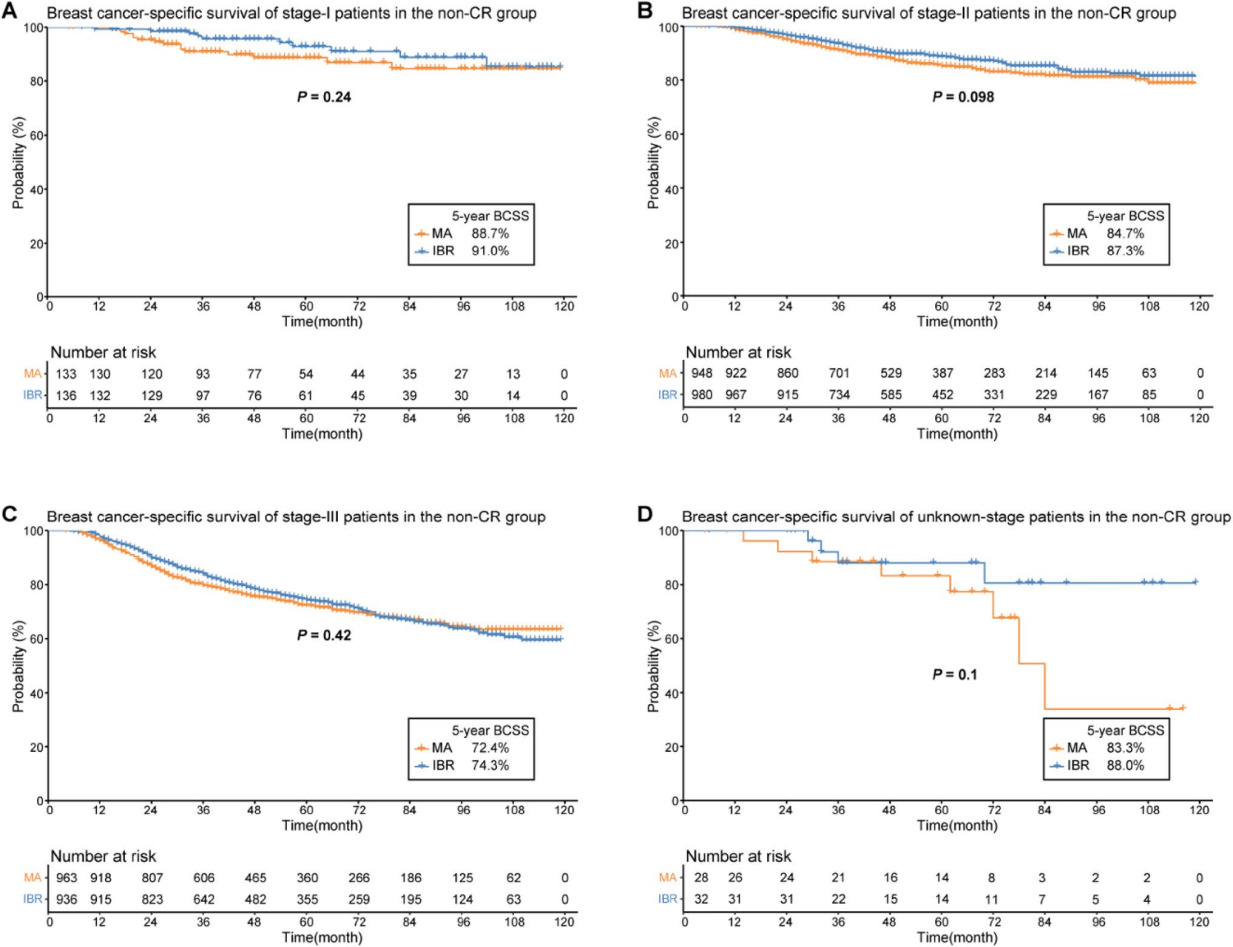
Breast cancer-specific survival rates of the MA versus IBR groups of the non-CR subgroup after propensity score matching. **A** Stage-I; **B** Stage-II; **C** Stage-III; **D** Unknown-stage. *BCSS* breast cancer-specific survival, *CR* complete response; *MA* mastectomy alone, *IBR* immediate breast reconstruction

The IBR group had a significantly higher 5-year BCSS rate prior to PSM (88.5 vs. 79.1%, *P* < 0.001) (Fig. 3). After PSM, a total of 9,610 individuals were enrolled for further survival analysis (Table 1). Kaplan–Meier curves with log-rank tests revealed a significant between-group difference in the 5 year BCSS rate (87.3 vs. 84.7%, *P* = 0.004) (Fig. 4). According to subgroup analysis, the 5 year BCSS rate was higher in the IBR group of the non-CR group (82.5 vs. 79.4%, *P* = 0.034) (Fig. 5), but did not differ in the CR or the unknown-response groups. There were no statistically significant between-group differences across the various stages after further stratification by the AJCC stage.

## Discussion

To our knowledge, the current work was the first SEER database-based research on the application of IBR for post-NAT patients. Our study showed a steady increase in IBR for post-NAT patients over the past few years. Meanwhile, IBR was proper and worthy of consideration for post-NAT patients, which might improve the 5-year BCSS.

Several reasons gave rise to the increase of IBR in the setting of NAT. On the one hand, the use of NAC has gradually increased since 2010, of which the aim gradually expanded from breast conservation to personalized treatment [18, 19]. The population base of our study kept growing for this reason. On the other hand, the Women's Health and Cancer Rights Act, which was passed in the US in 1998, mandated comprehensive coverage for breast reconstruction following mastectomy. An earlier study showed that the percentage of women getting breast reconstruction increased steadily over time [20]. Furthermore, several studies investigated the application of breast reconstruction in patients who were traditionally considered at high risk, and had achieved acceptable outcomes, which further improved the increase of IBR [21–23].

Before-PSM comparison showed IBR following NAT was associated with younger age, married status, earlier tumor stage and higher income, which were consistent with previous researches [11, 24, 25]. Analysis of prognosis showed the IBR was associated with improved BCSS. However, this beneficial association should be cautious, although several small-sample, single-center studies had demonstrated the feasibility and safety of IBR following NAT [9, 10, 26]. IBR lowered breast cancer-specific mortality in a prior sizable population-based trial, according to Bezuhly M et al. However, they also observed that these results were due to imbalances in socioeconomic factors and access to care rather than insufficient adjustment for tumor features and disease severity [27]. When the confounding effect of family income was taken into account, another study showed that immediate postmastectomy reconstruction had little benefits for survival [28]. In contrast, the previous Korean nationwide study confirmed that IBR following mastectomy was associated with worse prognoses than mastectomy alone among patients with advanced clinical stage tumor of the non-pCR group [11].

Although PSM was performed in this present work, some confounding factors were still unbalanced due to their unavailability in the SEER database such as baseline health status. Patients at a poor health status such as obesity, cardiovascular disease, and smoking history might not consider the IBR, but were associated with worse prognosis. We believe this potential imbalance caused the improved 5-year BCSS in the non-CR group. Additionally, the impact of IBR on personal life quality and psychology should also be considered. A previous meta-analysis demonstrated a better health-related quality of life (HRQOL) outcomes of breast reconstruction [29]. Another study revealed that reconstruction following a mastectomy significantly affects body image and sexual function [30]. Furthermore, a recent study demonstrated that poor HRQOL might increase the mortality risk [31]. Some patients with inoperable breast cancer in our study received NAT for breast conservation or reconstruction purpose, but might failed because of the severity of the disease (non-CR), which caused anxiety and depression. This potential psychological fluctuation during the treatment was a probable adverse factor, which led to a worse HRQOL with worse prognosis. However, in the absence of relevant data in the SEER database, we were unable to investigate the impact of personal life quality and psychology. Further researches to estimate HRQOL of the patients who received breast reconstruction after NAT are required.

There are several limitations in the use of the SEER database. Firstly, selection bias was inevitable in this retrospective study. Secondly, due to the lack of data on local recurrence, adequate evaluation of the oncology outcome is limited. Meanwhile, we are unable to access the perioperative complications among different treatments because of the unavailable data. Finally, this work was only based on population in the United States, and more real-word studies in other countries or regions are expected. Nonetheless, our retrospective work is able to provide some valuable reference in the absence of randomized controlled trials.

## Conclusion

The application of IBR in post-NAT patients has steadily increased. In the CR group, survival outcomes of post-NAT patients who received IBR were similar to those who received mastectomy alone. In the non-CR group, IBR was associated with potential survival benefits. More studies are expected to validate our findings.

## Data Availability

The datasets generated and analyzed during the current study are available from the corresponding author on reasonable request.
